# Isolation and Synthesis of Misszrtine A: A Novel Indole Alkaloid From Marine Sponge-Associated *Aspergillus* sp. SCSIO XWS03F03

**DOI:** 10.3389/fchem.2018.00212

**Published:** 2018-06-13

**Authors:** Rong Zhou, Xiaojian Liao, Hangbin Li, Jing Li, Pengju Feng, BingXin Zhao, Shihai Xu

**Affiliations:** Department of Chemistry, Jinan University, Guangzhou, China

**Keywords:** indole alkaloid, marine-derived fungi, secondary metabolites, sponge, total synthesis, antagonistic activity

## Abstract

A novel indole alkaloid, misszrtine A (**1**), was isolated from marine sponge-derived fungus *Aspergillus* sp. SCSIO XWS03F03. The planar structure of **1** was assigned by analysis of spectroscopic data, the absolute configuration of which was unambiguously determined by total synthesis. Compound **1** represents the first example of *N*-isopentenyl tryptophan methyl ester with a phenylpropanoic amide arm, which exhibited a potent antagonistic activity on HL60 (IC_50_ = 3.1 μM) and LNCaP (IC_50_ = 4.9 μM) cell lines. Bioactivity evaluation reveals that functional group on indole nitrogen of **1** has a great effect on its cytotoxity, which provides a mean to probe the structure-activity relationships of **1**.

## Introduction

Marine-derived fungi, a rich source of structurally diverse secondary metabolites, have attracted considerable attention due to their potential for the discovery of pharmaceutically interesting molecules (Attaway and Zaborsky, 1993; Newman and Cragg, 2016). Important drugs, such as Plinabulin, sorbicillactone A, etc., are derived from Marine-derived fungi (Bringmann et al., 2003; Nogueira et al., 2010; Millward et al., 2012). The genus *Aspergillus* (Moniliaceae) which firstly described by Micheli in 1729 was one of the largest and mostly intensively investigated fungal genera (Saleem et al., 2007; Ebrahim et al., 2016). A wide array of bioactive secondary metabolites from marine-derived *Aspergillus* species have been elucidated, including polyketides, terpenoids, alkaloids sterols, and peptides (Debbab et al., 2012), most of which exhibited antibacterial, antioxidant, radical-scavenging, cytotoxic, and anti-inflammatory activities (Li et al., 2004, 2012; Almeida et al., 2010; Lee et al., 2011; Sun et al., 2011).

**Figure 1:**
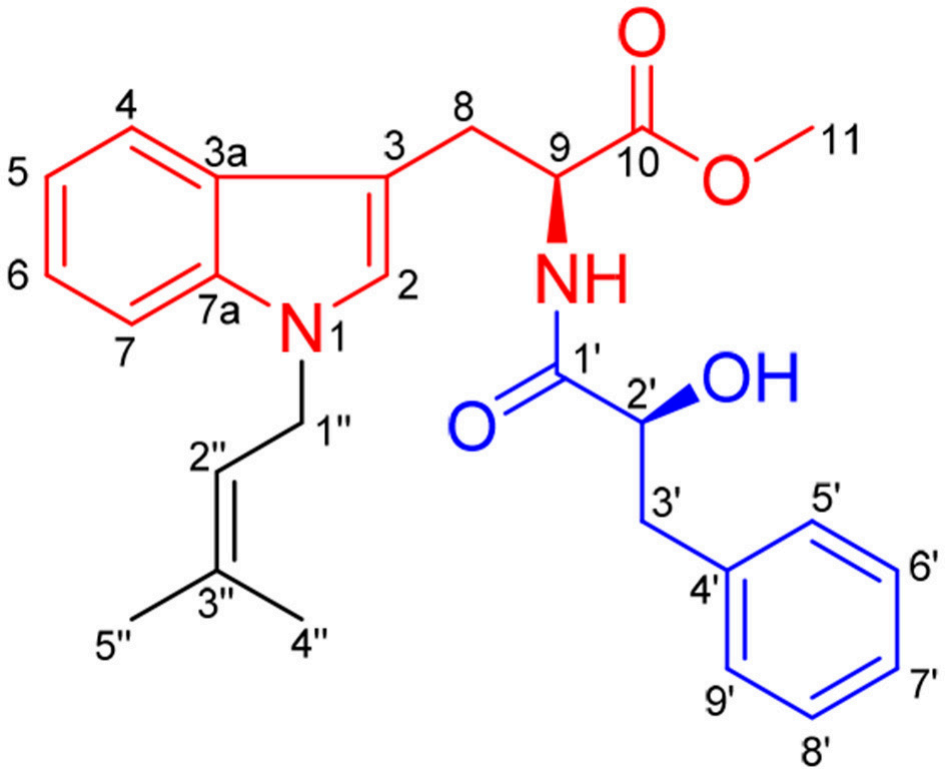
Chemical structure of misszrtine A (**1**).

The secondary metabolites from the sponge-derived fungus *Aspergillus* usually are terpenoids and polyketides. The alkaloids with an indole unit were extremely rare from the isolated structures (Varoglu et al., 1997). Previous investigations into marine sponge-associated fungus *Aspergillus* sp. SCSIO XWS03F03 by our group have resulted in the isolation of three polyketides, aspergchromones A, B, and secalonic acid D, along with other four small molecules (Wang Y. et al., 2016). In the course of our ongoing research on this fungus, a novel indole alkaloid, misszrtine A (**1**), was isolated by further investigation of the less polar fraction of the fungus extract. Compound **1** represents the first example of *N*-isopentenyl tryptophan methyl ester with a phenyl propanoic amide arm (Figure 1). Herein, we report the isolation, structural elucidation, plausible biogenetic route, total synthesis, and biological evaluation of **1**.

## Materials and methods

### General experimental procedures

Optical rotations were measured by using Anton Paar MCP-500 polarimeter (Auton Paar, Graz, Austria). UV spectra were recorded on a Shimadzu UV-2600 UV–vis spectrophotometer (Shimadzu, Kyoto, Japan). The NMR spectra were measured on a Bruker Av 300 or 500 MHz NMR spectrometer (Bruker, Fallanden, Switzerland). HR-ESI-MS were recorded on a Bruker micro TOF-QII mass spectrometer (Bruker, Fallanden, Switzerland). CD spectra were measured with a Chirascan circular dichroism spectrometer (JASCO International Co. Ltd., Hachioji, Tokyo, Japan). Size exclusion chromatography was done on Sephadex LH-20 gel (GE Healthcare, Uppsala, Sweden). Semi-preparative reversed-phase HPLC [Rp-C18: 9.4. 250 mm i.d, 5 μm (Cosmosil, Nacalai, Japan)] was performed on an Agilent 1200 series apparatus (Agilent, Palo Alto, American). Column chromatography (CC) was carried out on silica gel (200–300 mesh, Qingdao Marine Chemical Factory, Qingdao, China). Thin layer chromatography was carried out with precoated silica gel plates (GF-254, Jiangyou Silica Gel Development, Inc., Yantai, China).

### Fungal strain

The fungal strain SCSIO XWS03F03 was isolated from a sponge which was collected from the sea area Xuwen County, Guangdong Province, China, in August, 2013. The isolate was stored on MB agar (malt extract 15 g, sea salt 10 g, agar 15 g, distilled water 1,000 mL, pH 7.4~7.8) slants at 4°C and deposited at CAS Key Laboratory of Tropical Marine Bio-resources and Ecology.

### Its region sequence

The strain was cultured in Sabouraud's Dextrose Broth (consisting of 40 g dextrose, 10 g peptone, 2.5 g NaCl, and 1,000 mL distilled water, pH = 5.6) for 1.5 days, and then the mycelia were collected and powdered in a mixer mill after liquid nitrogen was added. DNA was isolated through the Hpure Fungal DNA Kit (Guangzhou Genebase Bioscience Co., Guangzhou, China) according to the manufacturer's protocol. The ITS region of strain SCSIO XWS03F03 was amplified by polymerase chain reaction with the primer pair ITS1-ITS4 (ITS1: 5′-GTA GTC ATA TGC TTG TCT C-3′; ITS4: 5′-TCC GCA GGT TCA CCT ACG GA-3′). The amplified product was purified with a TIANgel mini purification kit (TianGen Biotech, Beijing, China). The recovery pure PCR product was sequenced by a commercial service (Shanghai Majorbio Bio-pharm Technology Co., Ltd., Shanghai, China). Through BLAST-Algorithmus, the derived ITS region sequence was compared against the GenBank database (NCBI). Similarity analysis was performed by using Clustal W program (Nam et al., 2001).

### Nucleotide sequence accession number

The nucleotide sequence of the ITS region reported in this article was assigned the GenBank accession number KU975059.

### Fermentation, extraction, and isolation

Strain SCSIO XWS03F03 stored on MB agar slants at 4°C was cultured on MB agar plates and incubated at 25°C for 7 days. Seed medium (Melt extract 15 g, sea salt 10 g, distilled water 1,000 mL) was inoculated with strain SCSIO XWS03F03 and incubated at 25°C for 48 h on a rotating shaker (180 rpm; 25°C). Large scale fermentation in a solid rice medium of 1,000 mL flasks supplemented with 1% NaCl (rice 200 g, sea salt 2.0 g, distilled water 200 mL) (*n* = 45) was inoculated with 10 mL of seed solution. Flasks were incubated at 25°C under static condition and fermented for 45 days.

The fungal cultures from 45 flasks were harvested and the mycelia were cut into small pieces and soaked in acetone for 12 h, sonicated (10 min) and filtered, yielding the rice solid medium and water phases. The solid rice medium was extracted with ethyl acetate (EtOAc) (6 × 500 mL) and the water phase was extracted with EtOAc (3 × 10 L).Both EtOAc extracts were combined and partitioned with petroleum ether (PE) three times to remove the oil, then evaporated under vacuum at 42°C to yield 83 g crude extract, which was subjected to silica gel column chromatography (Φ 11 × 90 cm) eluting with PE/EtOAc in gradient eluent (90:10, 80:20, 70:30, 75:25, 65:35, 60:40, 50:50, 25:75, 0:100), to obtain 5 fractions (Fr. 1-5). Fr.2 (5.21 g) was then purified by silica gel column (PE/EtOAc, 90:10~0:100) to obtain six subfractions (Fr. 2-1~Fr. 2-6). Fr. 2-3 (256.3 mg) was further purified by semi preparative reversed-phase HPLC (2 mL/min, MeOH/H_2_O = 6/4) to give **1** (15 mg) (t_R_ = 20.3 min).

### Total synthesis of misszrtine a (1)

Methyl ((*S*)-2-hydroxy-3-phenylpropanoyl)-L-tryptophanate (**2**)

To a solution of L-3-Phenyllactic acid (4.0 g, 24.0 mmol) in dry degassed DCM (100 mL) was added EDCI (4.56 g, 24.0 mmol), HOBt (3.24 g, 24.0 mmol), and L-tryptophan methyl ester hydrochloride (5.1 g, 20.0 mmol). After stirring for 30 h, the reaction mixture was concentrated and extracted with EtOAc (20 mL × 2). The combined organic extracts were washed with saturated aqueous NaHCO_3_ (20 mL × 2), dried over MgSO_4_, filtered, concentrated, and purified by flash chromatography over 200–400 mesh silica gel (PE/EA = 4:1) to give **2** as a light yellow oil (6.38 g, 87%). ^1^H NMR (300 MHz, CDCl_3_) δ 8.96 (s, 1H), 7.53 (d, *J* = 7.6 Hz, 1H), 7.47 (d, *J* = 8.1 Hz, 1H), 7.41–7.17 (m, 8H), 6.73 (s, 1H), 5.99 (dd, *J* = 12.9, 5.2 Hz, 1H), 4.36 (dd, *J* = 6.2, 3.6 Hz, 1H), 3.93 (brs, 1H), 3.61 (s), 3.36 (dd, *J* = 14.7, 7.2 Hz, 1H), 3.24–3.10 (m, 2H), 2.91 (dd, *J* = 13.7, 7.2 Hz, 1H). ^13^C NMR (75 MHz, CDCl_3_) δ 173.5, 136.9, 136.0, 129.6, 128.2, 127.1, 126.6, 123.2, 121.8, 119.2, 118.2, 111.4, 108.7, 77.4, 72.3, 52.2, 40.1, 27.5. HRESIMS *m/z* 367.1625 [M+H] ^+^ (calcd for C_21_H_22_N_2_O_4_ 367.1652).

Methyl ((*S*)-2-((*tert*-butyldimethylsilyl)oxy)-3-phenylprop anoyl)-L-tryptophanate (**3**)

To a solution of **2** (1.83 g, 5.0 mmol) in dry MeCN (50 mL) was added imidazole (0.14 g, 2.0 mmol) and TBSCl (0.91 g, 6.0 mmol); After stirring for 2 h, the combined organic extracts were washed with saturated aqueous NaHCO_3_(10 mL × 2), dried over MgSO_4_, filtered, concentrated, and purified by flash chromatography over 200–400 mesh silica gel (PE/EA = 1:1) to give **3** as a yellow gum (2.30 g, 96%). ^1^H NMR (300 MHz, DMSO-d_6_) δ 10.9 (s, 1H), 7.35–7.23 (m, 5H), 7.19–7.12 (m, 2H), 7.05 (td, *J* = 8.1, 1.1 Hz, 1H), 6.96 (td, *J* = 8.1, 0.8 Hz, 1H), 6.83 (d, *J* = 2.3 Hz, 1H), 4.77–4.65 (m, 1H), 4.21 (dd, *J* = 7.5, 3.7 Hz, 1H), 3.58 (s, 3H), 3.18 (dd, *J* = 14.6, 5.9 Hz), 2.98 (dd, *J* = 14.6, 5.4 Hz, 1H), 2.88 (dd, *J* = 13.4, 3.6 Hz, 1H), 2.70 (dd, *J* = 13.4, 7.5 Hz, 1H), 0.61 (s, 9H), −0.25 (s, 3H), −0.33 (s, 3H); ^13^C NMR (75 MHz, DMSO-d_6_) δ 172.1, 171.9, 137.7, 136.6, 130.4, 128.3, 127.6, 126.8, 124.1, 121.4, 119.0, 118.4, 111.8, 108.5, 74.3, 52.4, 52.1, 41.4, 27.6, 25.8, 17.9, −5.3, −5.6.

Methyl((*S*)-2-((tert-butyldimethylsilyl)oxy)-3-phenylpropanoyl)-1-(3-methylbut-2-en-1-yl)-L-tryptophanate (**4**)

To a solution of **3** (0.96 g, 1.0 mmol) in dry DMF (50 mL) was added NaH (0.05 g, 2.0 mmol). After stirring for 1 h, 1-bromo-3-methyl-2-butene (0.3 mL, 1.5 mmol) was added dropwise to the reaction mixture. Upon stirring for 4 h, the reaction was quenched with water and extracted with EtOAc (10 mL × 3). The combined organic extracts were washed with saturated aqueous NaHCO_3_(10 mL × 2), dried over MgSO_4_, filtered, concentrated, and purified by flash chromatography over 200–400 mesh silica gel (PE/EA = 1:1) to give **4** as a yellow gum (0.99 g, 90%). ^1^H NMR (300 MHz, DMSO-d_6_) δ 7.38–7.20 (m, 6H), 7.20–7.15 (m, 2H), 7.11 (t, *J* = 7.9 Hz, 1H), 7.00 (t, *J* = 7.3 Hz, 1H), 6.73 (s, 1H), 5.24 (t, *J* = 6.7 Hz, 1H), 4.80–4.70 (m, 1H), 4.68–4.60 (m, 2H), 4.23 (dd, *J* = 7.2, 3.7 Hz, 1H), 3.58 (s, 1H), 3.20 (dd, *J* = 14.6, 5.8 Hz, 1H), 2.98 (dd, *J* = 14.6, 5.3 Hz, 1H), 2.88 (dd, *J* = 13.4, 3.7 Hz, 1H), 2.75 (dd, *J* = 13.4, 7.4 Hz, 1H), 1.79 (s, 3H), 1.68 (s, 3H), −0.23 (s, 3H), −0.31 (s, 3H).

Methyl-((*S*)-2-hydroxy-3-phenylpropanoyl)-1-(3-methylbut-2-en-1-yl)-L-tryptophanate (**1**)

To a solution of **3** (0.55 g, 1.0 mmol) in dry THF (30 mL) was added dropwise pyridine-HF (2.3 mL, 10.0 mmol). After stirring for 1 h, the reaction mixture was quenched with water and extracted with DCM (10 mL × 3). The crude product was purified by HPLC to give **1** as a white solid (0.40 g, 92%). [α]${}_{\text{D}}^{25}$ −5.35 (*c* 2, CH_3_OH); UV(MeOH) λ_max_ (log ε) 225 (1.51) nm 275(0.25)nm; ^1^H NMR (300 MHz, Chloroform-d) δ 7.42 (d, *J* = 7.9 Hz, 1H), 7.36–7.18 (m, 7H), 7.11 (d, *J* = 7.5 Hz, 1H), 7.06 (d, *J* = 6.8 Hz, 1H), 6.70 (s, 1H), 5.34 (t, *J* = 6.8 Hz, 1H), 4.97–4.90 (m, 1H), 4.64 (s, 1H), 4.62 (s, 1H), 4.30 (dd, *J* = 7.7, 4.1 Hz, 1H), 3.30 (dd, *J* = 14.7, 5.6 Hz, 1H), 3.20–3.10 (m, 2H), 2.88 (d, *J* = 7.7 Hz, 1H), 2.83 (d, *J* = 7.7 Hz,1H), 1.84 (s, 3H), 1.78 (s, 3H).^13^C NMR (75 MHz, Chloroform-d) δ 172.5, 172.2, 136.8, 136.2, 136.2, 129.8, 128.6, 128.2, 126.95, 126.2, 121.6, 119.95, 119.1, 118.7, 109.7, 108.2, 72.6, 52.5, 52.3, 44.1, 40.5, 27.8, 25.7, 18.1.

### Evaluation of general cytotoxicity of compound 1 and 2

Cytotoxic activity was assessed by using the CCK-8 (Dojindo, Japan) method according to established procedures (Bai et al., 2014). Eight human cancer cell lines (HepG2, HL60, Hela, A375, A549, HT29, SK-BR-3, LNCaP, and MCF-7) were used in the cytotoxicity bioassay. The cells were cultured in RPMI or DMEM medium supplemented with 10% heat-inactivated fetal bovine serum (Gibco, USA) with 5% CO_2_ in air at 37°C. A cell viability assay was determined with the CCK-8 (Dojindo, Japan) assay. Cells were seeded at a density of 400–800 cells/well in 384-well plates and treated with various concentrations of compounds or solvent control. After 72 h incubation, CCK-8 reagent was added, and absorbance was measured at 450 nm using an Envision 2104 multilabel reader (PerkinElmer, USA). Dose–response curves were plotted to determine the IC_50_ values by using Prism 5.0 (GraphPad Software Inc., USA).

## Results and discussion

### Identification of (*S, S*)-misszrtine A

Misszrtine A (**1**) was obtained as a light yellow powder, which showed a molecular ion cluster [M+H]^+^ at 435.2284, revealing a molecular formula C_26_H_30_N_2_O_4_, indicating thirteen degrees of unsaturation. The UV absorption maximum at 225 and 290 nm implied the presence of an indole chromophore in **1** (Sangster and Stuart, 1995). The IR bands implied the presence of amino group (3380 cm^−1^) hydroxyl group (3262 cm^−1^), carbonyl groups (1745 and 1731 cm^−1^), and aromatic ring (1645 and 1535 cm^−1^). The analysis of NMR spectra revealed the presence of two carbonyls (δ_C_ 172.2 and 172.1), a monosubstituted benzene ring [δ_H_ 7.29 (2H), 7.26 (1H), and 7.23 (2H); δ_C_ 136.6, 129.7, 128.7, and 127.0], a disubstituted benzene ring [δ_H_ 7.37 (1H, dd, *J* = 7.8, 1.2 Hz), 7.27 (1H), 7.18 (1H, ddd, *J* = 7.2, 6.9, 1.2 Hz), and 7.06 (1H, ddd, *J* = 7.8, 6.9, 1.2 Hz); δ_C_ 136.2, 128.2, 121.6, 119.1, 118.7, and 109.6], two trisubstituted double bonds [δ_H_ 5.30 (1H, m) and 6.69 (1H, s); δ_C_ 136.3, 126.1, 119.9, and 108.2), two methines [δ_H_ 4.92 (1H, ddd, *J* = 8.4, 5.4, 5.1 Hz) and 4.29 (1H, dd, *J* = 7.8, 4.2 Hz); δ_C_ 72.7 and 52.5], three methenes [δ_H_ 4.60 (2H, d, *J* = 6.9 Hz), 3.28 (1H, dd, *J* = 14.7, 5.4 Hz), 3.17 (1H, dd, *J* = 14.7, 5.1 Hz), 3.13 (1H, dd, *J* = 13.8, 4.2 Hz), and 2.83 (1H, dd, *J* = 13.8, 7.8 Hz); δ_C_ 44.0, 40.5, and 27.8], a methoxyl [δ_H_ 3.66 (1H, s); δ_C_ 52.3] and two methyls [δ_H_ 1.80 (3H, d, *J* = 1.2Hz) and 1.74 (3H, d, *J* = 1.2Hz); δ_C_ 25.6 and 18.0]. The above spectral data suggested that misszrtine A (**1**) might be an indole alkaloid (Estevão et al., 2010; Khalil et al., 2014). A comprehensive analysis of the ^1^H-^1^H COSY, HSQC, and HMBC spectra allowed the full assignment of all proton and carbon resonances of **1** as shown in Table 1.

**Table 1:** ^1^H and ^13^C NMR spectral data of **1** (*J* in Hz, in CDCl**3**)^*a*^.

**No**.	**Misszrtine A (1)**	
	**δ_H_**	**δ_C_**
2	6.69 (s)	126.1
3	–	108.2
3a	–	128.2
4	7.37 (dd, 7.8, 1.2)	118.7
5	7.06 (ddd, 7.8, 6.9, 1.2)	119.1
6	7.18 (ddd, 7.2, 6.9, 1.2)	121.6
7	7.27	109.6
7a	–	136.2
8	a 3.28 (dd, 14.7,5.4)	27.8
	b 3.17 (dd, 14.7, 5.1)	
9	4.92 (ddd, 8.4, 5.4, 5.1)	52.5
10	–	172.1
11	3.66 (s)	52.3
1′	–	172.2
2′	4.29 (dd,7.8,4.2)	72.7
3′	a 3.13 (dd,13.8,4.2)	40.5
	b 2.83(dd,13.8, 7.8)	
4′	–	136.6
5′	7.23	129.7
6′	7.29	128.7
7′	7.26	127.0
8′	7.29	128.7
9′	7.23	129.7
1″	4.60 (d,6.9)	44.0
2″	5.30 (m)	119.9
3″	–	136.3
4″	1.80 (d, 1.2)	18.0
5″	1.74 (d, 1.2)	25.6
NH	6.95 (d, 8.4)	−

a *Overlapped signals are reported without designating multiplicity*.

**Figure 2:**
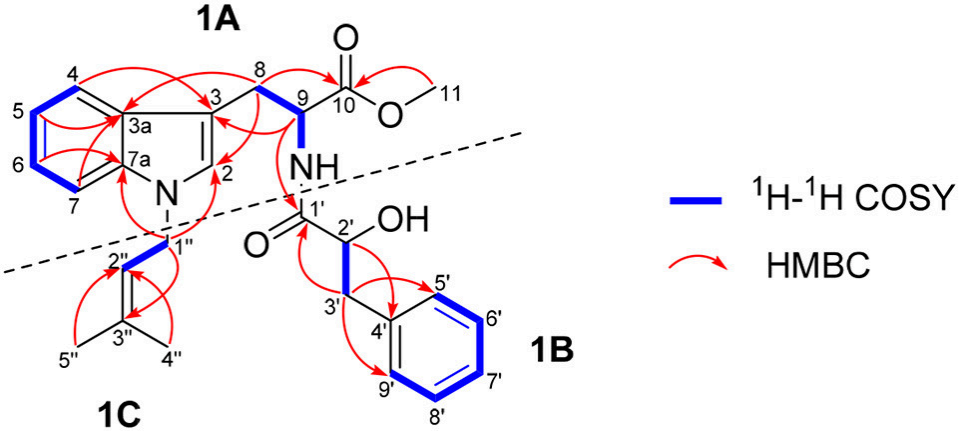
Key ^1^H-^1^H COSY and HMBC correlations of **1**.

The ^1^H-^1^H COSY data of misszrtine A (**1**) revealed the presence of five spin coupling systems in bold as shown in Figure 2. In the HMBC spectrum, correlations between H-4/H-9 and C-3, between H-5/H-7/H-8 and C-3a, between H-6 and C-7a, between H-8 and C-2/C-10 as well as between H-11 and C-10 allowed the establishment of tryptophanmethyl ester moiety (**1A**). Furthermore, the HMBC correlations from H-3′ to C-1′/C-5′/C-9′ as well as from H-2′ to C-4′ validated the presence of phenylpropanoic acid unit. Additionally, according to the molecular formula information and the obvious downfield shifts at C-2′, a hydroxyl group should be attached to C-2′. Thus, the structure of α-hydroxy-phenylpropanoic acid residue (**1B**) was deduced. Moreover, the HMBC correlations between H-1″ and C-3″ as well as between H-4″/H-5″ and C-2″ verified the skeleton of isopentene group (**1C**). In addition, the HMBC correlations between H-9 and C-1′ suggested that **1A** and **1B** were connected via N-C-1′ bond. The HMBC correlations from H-1″ to C-2/C-7a indicated that **1C** was linked to N-1. Therefore, the planar structure of **1** was identified (Figure 2).

**Scheme 1:**
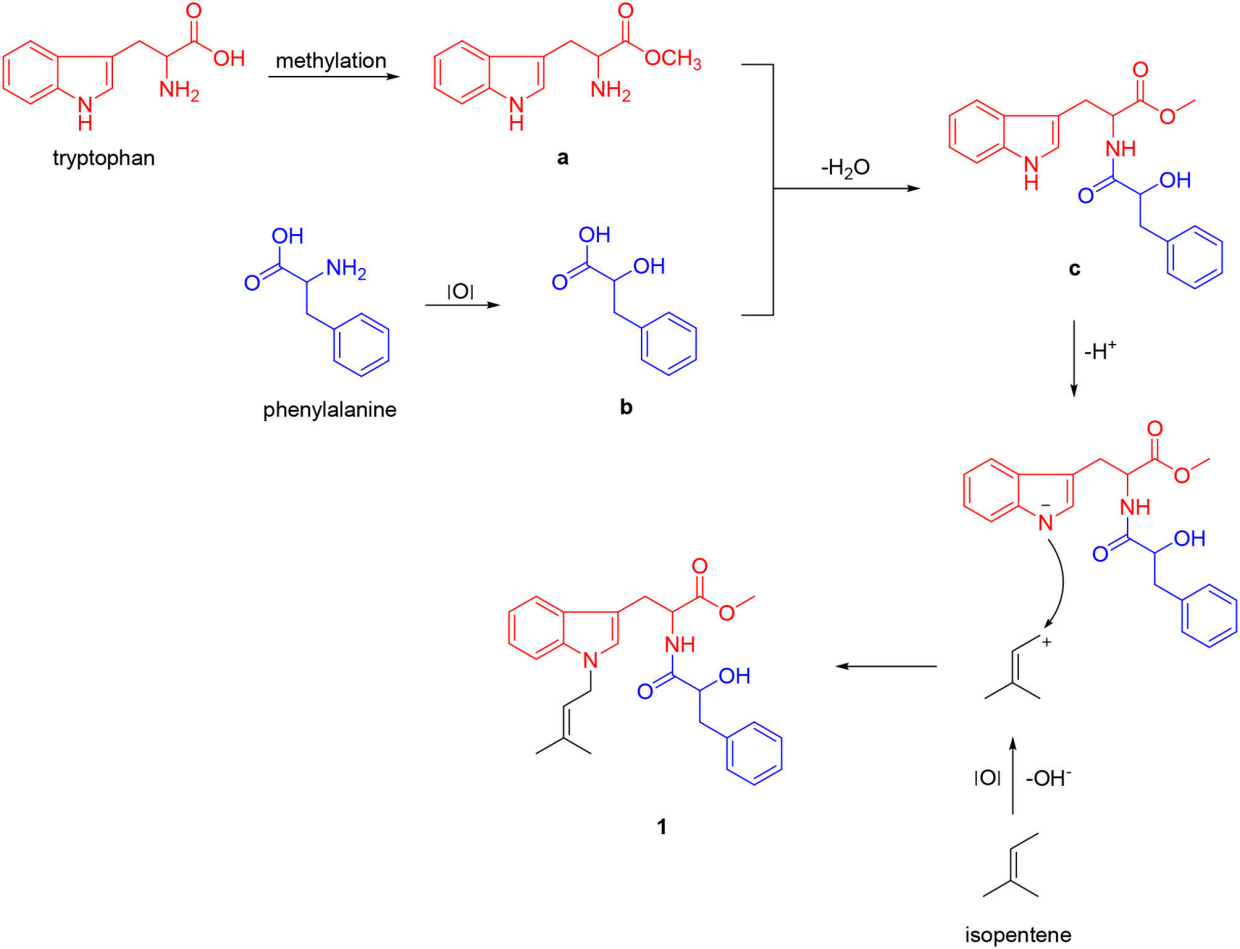
Plausible biogenetic route of **1**.

**Scheme 2:**
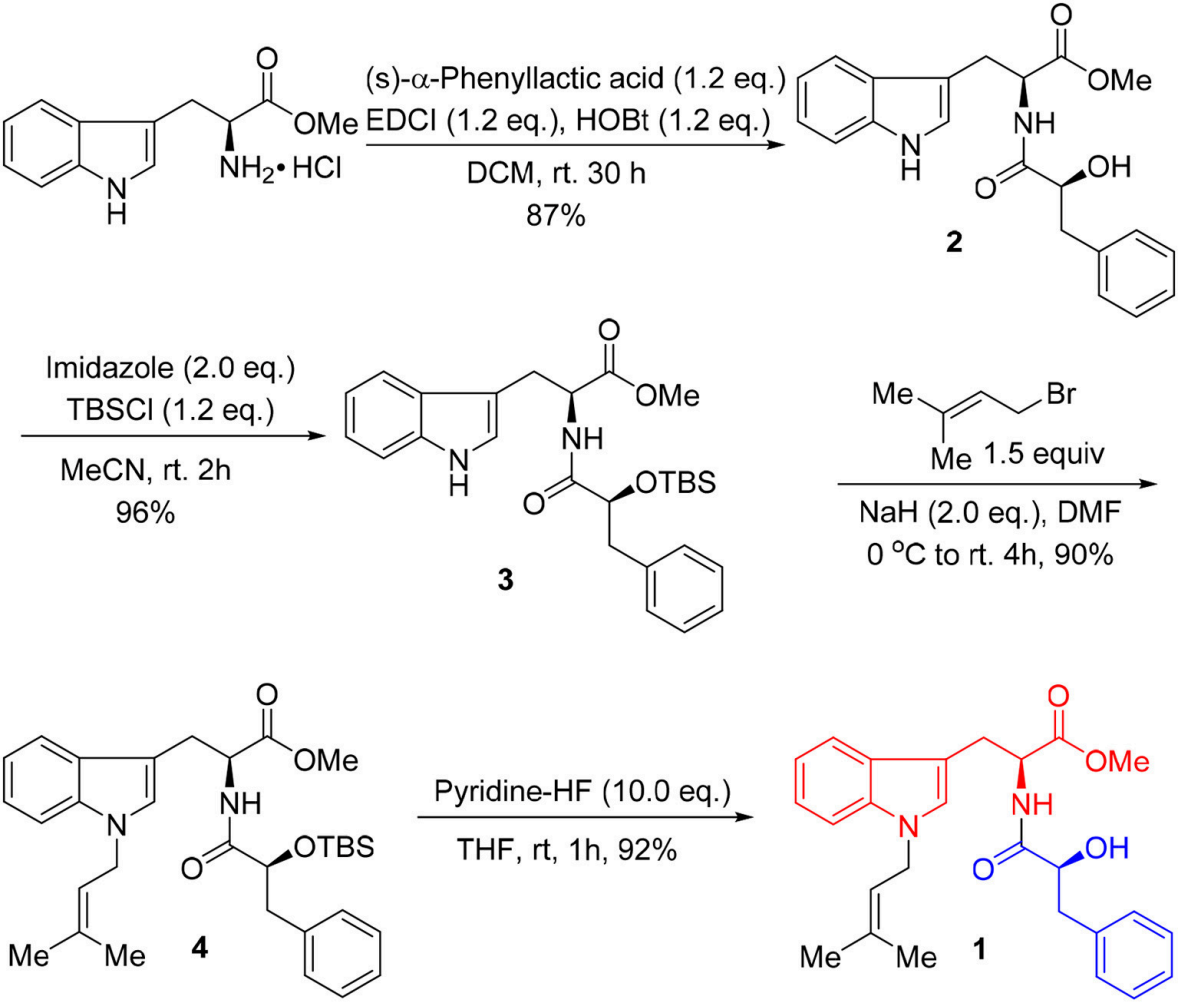
Synthetic route of misszrtine A (**1**).

Unfortunately, no helpful NOE correlation was observed in the NOESY spectrum to determine the relative stereochemistry of **1**. However, the biogenetic route of **1** could be proposed, which was plausibly traced back to tryptophan, phenylalanine, and isopentene. First, tryptophan was methylated to form tryptophan methyl ester (**a**). Meanwhile, phenylalanine was oxidized to α-hydroxy-3-phenylpropanoic acid (**b**) (Winitz et al., 1956; Khelifa et al., 1998; Senkpeil et al., 2002; Busto et al., 2014). Intermediates **a** and **b** were dehydrated to give compound **c**, which took a nucleophilic substitution reaction with isopentene to produce **1** (Scheme 1). Accordingly, a total synthesis of **1** was carried out to confirm the absolute configurations of C-9 and C-2′ (Scheme 2). Since the L configuration of α-amino acids are more common in nature, L-tryptophan methyl ester hydrochloride and (*S*)-α-hydroxy-3-phenylpropanoic acid were selectively chosen as substrates to undergo an amidation coupling, yielding compound **2** (87% yield) with two chiral centers (Airiau et al., 2008). The hydroxyl group was chemo-selectively protected by TBSCl to give compound **3** in 96% yield. After removing N-H proton of **3** with NaH at 0°C, bromo-3-methyl-2-butene was injected to the reaction mixture, furnishing a protected indole **4** in 90% yield without any racemic counterparts (Estevão et al., 2010). Pyridine-HF reagent (Ohshima et al., 2003; Shiozaki et al., 2013) was employed to remove TBS group under slightly acidic condition for delivering compound **1** which was then fully characterized.

**Figure 3:**
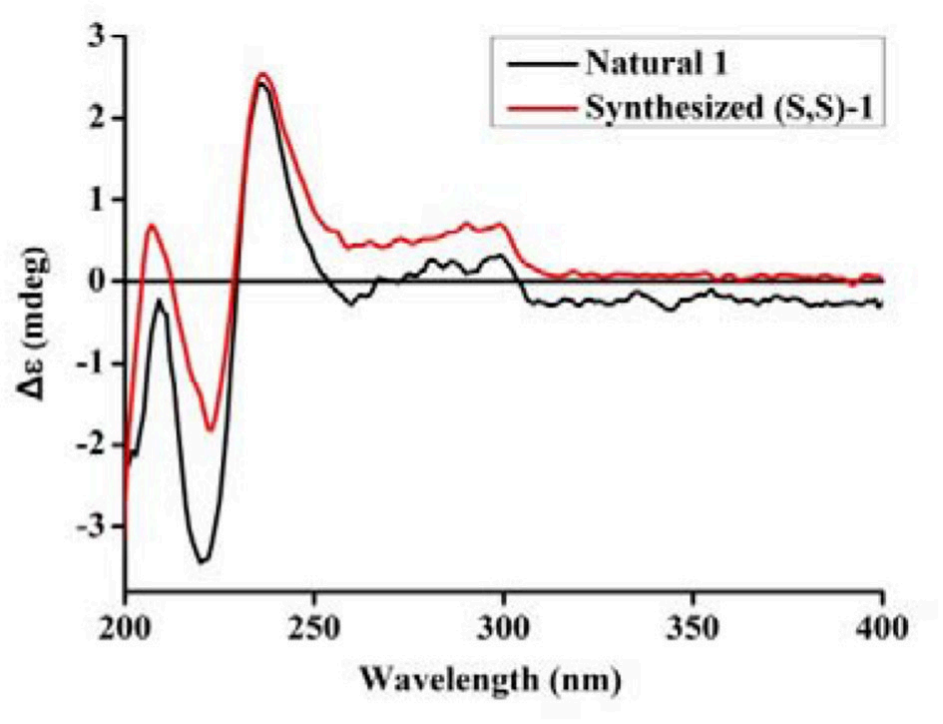
CD spectrum of synthesized (*S*,*S*)-**1** and natural **1**.

The UV spectrum, NMR data, optical rotations, and CD profiles of the natural misszrtine A (**1**) were compared with those of synthesized (*S, S*)-**1**. As shown in supporting information, the UV spectrum, ^1^H and ^13^C NMR data for natural **1** were essentially identical to those of synthesized (*S, S*)-**1**. As for specific optical rotation, the value of natural Misszrtion A ([α]${}_{\text{D}}^{25}$-6.13 (C 0.10, CH_3_OH)) was in good agreement with that of the synthesized (*S, S*)-**1** ([α]${}_{\text{D}}^{25}$-5.35 (C 2.0, CH_3_OH)), indicating that the absolute configuration at both C-9 and C-2″of natural misszrtine A (**1**) were *S*. In addition, the results from CD profiles showed that natural misszrtine A (**1**) had identical pattern as that of synthesized (*S, S*)-**1** (Figure 3).

### Biological evaluation of misszrtine A (1) and compound 2

The synthesized key intermediate **2** and misszrtine A (**1**) were selected for the evaluation of general cytotoxicity toward nine human cancer cell lines (HepG2, HL60, Hela, A375, A549, HT29, SK-BR-3, LNCaP, and MCF-7) (Table 2). Misszrtine A (**1**) exhibited cytotoxic activity against HL-60 and LNCap cells with IC_50_ values of 3.1 and 4.9 μM, respectively, but with no cytotoxicity against the rest of tested cell lines. Interestingly, compound **2**, without protecting group on the indole nitrogen, showed a lost cytotoxicity toward HL-60 and LNCap cell lines, indicating that the nitrogen protecting group may be a key factor for tuning biological activities of misszrtine A (**1**) derivatives. Misszrtine A (**1**) was also tested for inhabitation against H3N2 influenza, antituberculous activity, anti-inflammatory activity using COX-1 and COX-2 as molecular targets (Wang J. et al., 2016). However, no positive results were obtained thus far.

**Table 2:** Biological evaluation of misszrtine A (**1**) and compound **2**^*a*^.

**Cmpd**	**Observed IC**_**50**_ **(**μ**M)**								
	**HepG2**	**HL-60**	**Hela**	**A375**	**A549**	**HT29**	**SK-BR-3**	**LNCap**	**MCF-7**
**1**	NA	3.12	NA	NA	>30	>30	>30	4.94	>30
**2**	NA	>30	NA	NA	NA	NA	NA	NA	NA

a *Antitumor activities were determined by microdilution method. “>30 μM” means cell survival rate between 70–85%; “NA” means cell survival rate over 85%*.

## Conclusion

In summary, a novel indole alkaloid (misszrtine A, **1**), with two chiral centers, was isolated and characterized from the marine sponge-derived *Aspergillus* sp. SCSIO XWS03F03. The planar structure of **1** was assigned by analysis of UV, IR, and NMR spectroscopic data, and its absolute configuration was unambiguously determined by total synthesis and specific optical rotation, CD spectrum comparison. Bioactivity evaluation results showed that compound **1** exhibited a potent antagonistic activity on HL60 (IC_50_ = 3.1 μM) and LNCaP (IC_50_ = 4.9 μM) cell lines, while compound **2** was inactive to those cell lines. The findings indicated that functional group on indole nitrogen of **1** has a great effect on its cytotoxity, which provides a mean to probe the structure-activity relationships of **1**.

## Author contributions

RZ and XL contributed equally. RZ was responsible for the isolation and synthesis of misszrtine A. XL did the purification and identification of compound. HL, JL, and BZ are responsible for the fermentation and extraction of the fungal strain. PF was designed the synthesis. BZ, PF, and SX were prepared the manuscript, and all authors have approved the final version.

### Conflict of interest statement

The authors declare that the research was conducted in the absence of any commercial or financial relationships that could be construed as a potential conflict of interest.

## References

[B1] Airiau E. Spangenberg T. Girard N. Schoenfelder A. Salvadori J. Taddei M.. (2008). A general approach to Aza-Heterocycles by means of domino sequences driven by hydroformylation. Chem. Eur. J. 14, 10938–10948. 10.1002/chem.200801795 19009576

[B2] Almeida A. P. Dethoup T. Singburaudom N. Lima R. Vasconcelos M. H. Pinto M. (2010). The *in vitro* anticancer activity of the crude extract of the sponge-associated fungus Eurotium cristatum and its secondary metabolites. J. Nat. Pharm. 1, 25–29. 10.4103/2229-5119.73583

[B3] Attaway D. H. Zaborsky O. R. (eds). (1993). Marine Biotechnology. Pharmaceutical and Bioactive Natural Products, Vol. 1. New York, NY: Plenum Press.

[B4] Bai Z.-Q. Lin X. Wang Y. Wang J. Zhou X. Yang B.. (2014). New phenyl derivatives from endophytic fungus *Aspergillus flavipes* AIL8 derived of mangrove plant *Acanthus ilicifolius*. Fitoterapia 95, 194–202. 10.1016/j.fitote.2014.03.021 24704337

[B5] Bringmann G. Lang G. Muhlbacher J. Schaumann K. Steffens S. Rytik P. G.. (2003). Sorbicillactone A: a structurally unprecedented bioactive novel-type alkaloid from a sponge-derived fungus. Prog. Mol. Subcell. Biol. 37, 231–253. 10.1007/978-3-642-55519-0_9 15825646

[B6] Busto E. Richter N. Grischek B. Kroutil W. (2014). Biocontrolled formal inversion or retention of L-α-amino acids to enantiopure (R)- or (S)-hydroxyacids. Chemistry 20, 11225–11228. 10.1002/chem.201403195 25048982

[B7] Debbab A. Aly A. H. Proksch P. (2012). Endophytes and associated marine derived fungi—ecological and chemical perspectives. Fungal Divers. 57, 45–83. 10.1007/s13225-012-0191-8

[B8] Ebrahim W. El-Neketi M. Lewald L. I. Orfali R. S. Lin W. Rehberg N.. (2016). Metabolites from the fungal endophyte *Aspergillus austroafricanus* in axenic culture and in fungal-bacterial mixed cultures. J. Nat. Prod. 79, 914–922. 10.1021/acs.jnatprod.5b00975 27070198

[B9] Estevão M. Carvalho L. C. Ribeiro D. Couto D. Freitas M. Gomes A.. (2010). Antioxidant activity of unexplored indole derivatives: synthesis and screening. Eur. J. Med. Chem. 45, 4869–4878. 10.1016/j.ejmech.2010.07.059 20727623

[B10] Khalil Z. G. Huang X.-C. Raju R. Piggott A. M. Capon R. J. (2014). Shornephine A: structure, chemical stability, and P-glycoprotein inhibitory properties of a rare diketomorpholine from an Australian marine-derived Aspergillus sp. J. Org. Chem. 79, 8700–8705. 10.1021/jo501501z 25158286 PMC4168782

[B11] Khelifa N. Butel M.-J. Rimbault A. (1998). Synthesis of 2-hydroxy acid from 2-amino acid by *Clostridium butyricum*. Bioorg. Med. Chem. Lett. 8, 3429–3434. 10.1016/S0960-894X(98)00620-9 9873747

[B12] Lee D. S. Jeong G. S. Li B. Lee S. U. Oh H. Kim Y. C. (2011). Asperlin from the marine-derived fungus Aspergillus sp. SF-5044 exerts anti-inflammatory effects through heme oxygenase-1 expression in murine macrophages. J. Pharmacol. Sci. 116, 283–295. 10.1254/jphs.10219FP 21705844

[B13] Li D. Xu Y. Shao C. L. Yang R. Y. Zheng C. J. Chen Y. Y.. (2012). Antibacterial bisabolane-type sesquiterpenoids from the sponge-derived fungus Aspergillus sp. Mar. Drugs 10, 234–241. 10.3390/md10010234 22363233 PMC3280534

[B14] Li Y. Li X. F. Kim S. K. Kang J. S. Choi H. D. Rho J. R.. (2004). Golmaenone, a new diketopiperazine alkaloid from the marine-derived fungus Aspergillus sp. Chem. Pharm. Bull. 52, 375–376. 10.1248/cpb.52.375 14993767

[B15] Millward M. Mainwaring P. Mita A. Federico K. Lloyd G. K. Reddinger N.. (2012). Phase 1 study of the novel vascular disrupting agent plinabulin (NPI-2358) and docetaxel. Invest. New Drugs 30, 1065–1073. 10.1007/s10637-011-9642-4 21327495

[B16] Nam J.-W. Nojiri H. Yoshida T. Habe H. Yamane H. Omori T. (2001). New classification system for oxygenase components involved in ring-hydroxylating oxygenations. Biosci. Biotechnol. Biochem. 65, 254–263. 10.1271/bbb.65.254 11302156

[B17] Newman D. J. Cragg G. M. (2016). Drugs and drug candidates from marine sources: an assessment of the current “State of Play”. Planta Med. 82, 775–789. 10.1055/s-0042-101353 26891002

[B18] Nogueira I. Lobo-da-Cunha A. Afonso A. Rivera S. Azevedo J. Monteiro R. (2010). Toxic effects of domoic acid in the seabream *Sparus aurata*. Mar. Drugs 8, 2721–2732. 10.3390/md8102721 21116416 PMC2993002

[B19] Ohshima T. Gnanadesikan V. Shibuguchi T. Fukuta Y. Nemoto T. Shibasaki M. (2003). Enantioselective syntheses of aeruginosin 298-A and its analogues using a catalytic asymmetric phase-transfer reaction and epoxidation. J. Am. Chem. Soc. 125, 11206–11207. 10.1021/ja037290e 16220936

[B20] Saleem M. Ali M. S. Haussain S. Jabbar A. Ashraf M. Lee Y. S. (2007). Marine natural products of fungal origin. Nat. Prod. Rep. 24, 1142–1152. 10.1039/b607254m 17898901

[B21] Sangster A. W. Stuart K. L. (1995). Ultraviolet spectra of alkaloids. Chem. Rev. 65, 69–130. 10.1021/cr60233a003

[B22] Senkpeil R. F. Pantaleone D. P. Taylor P. P. (2002). Production of Hydroxyl-Carboxylic Acids Using a Coupled Enzyme System. Chicago, IL. WO 2002/033110.

[B23] Shiozaki M. Tashiro T. Koshino H. Shigeura T. Watarai H. Taniguchi M.. (2013). Synthesis and biological activity of hydroxylated analogues of KRN7000 (α-galactosylceramide). Carbohydr. Res. 370, 46–66. 10.1016/j.carres.2013.01.010 23454137

[B24] Sun H. H. Mao W. J. Jiao J. Y. Xu J. C. Li H. Y. Chen Y.. (2011). Structural characterization of extracellular polysaccharides produced by the marine fungus Epicoccumnigrum JJY-40 and their antioxidant activities. Mar. Biotechnol. 13, 1048–1055. 10.1007/s10126-011-9368-5 21279405

[B25] Varoglu M. Corbett T. H. Valeriote F. A. Crews P. (1997). Asperazine, a selective cytotoxic alkaloid from a sponge-derived culture of *Aspergillus niger*. J. Org. Chem. 62, 7078–7079. 10.1021/jo970568z 11671801

[B26] Wang J. Wei X. Qin X. Tian X. Liao L. Li K.. (2016). Antiviral merosesquiterpenoids produced by the antarctic fungus *Aspergillus ochraceopetaliformis* SCSIO 05702. J. Nat. Prod. 79, 59–65. 10.1021/acs.jnatprod.5b00650 26697718

[B27] Wang Y. Lin X.-P. Lu Z.-R. Liao X.-J. Huang X.-J. Zhang C.. (2016). Aspergchromones A and B, two new polyketides from the marine sponge-associated fungus Aspergillus sp. SCSIO XWS03F03. J. Asian Nat. Prod. Res. 19, 684–690. 10.1080/10286020.2016.1231673 28276769

[B28] Winitz M. Frankenthal L. B. Lzumiya N. Birnbaum S. M. Baker C. G. Greenstein J. P. (1956). Studies on diastereoisomeric α-amino acids and corresponding α-hydroxy acids VI.rotatory dispersion of copper complexes. J. Am. Chem. Soc. 78, 1602–1605. 10.1021/ja01589a027

