# Supplementary material for: Isolation and Synthesis of Misszrtine A: A Novel Indole Alkaloid From Marine Sponge-Associated Aspergillus sp. SCSIO XWS03F03
Source: Front Chem. 2018 Jun 13;6:212. doi: 10.3389/fchem.2018.00212 (PMC6008316; doi:10.3389/fchem.2018.00212)

Supporting Information

Isolation and Synthesis of Misszrtine A: a Novel Indole Alkaloid from Marine Sponge-associated *Aspergillus* sp. SCSIO XWS03F03

Rong Zhou,‡ Xiaojian Liao,‡ Hangbin Li, Jing Li, Pengju Feng*, Bingxin Zhao* and Shihai Xu*

Department of Chemistry, Jinan University, Guangzhou 510632, China

* E-mail: pfeng@jnu.edu.cn;
zbx840622@163.com;
txush@jnu.edu.cn

Contents

Natural Misszrtine A (**1**)....................2
Total synthesis of Misszrtine A (**1**):....................2
Figure S1. UV spectrum of MisszrtlideA (**1**) in MeOH. 3
Figure S2. CD spectrum of MisszrtlideA (**1**) in MeOH. 3
NMR spectra: 4

Natural Misszrtine A (1)

methyl *N*-((*S*)-2-hydroxy-3-phenylpropanoyl)-1-(3-methylbut-2-en-1-yl)-L-tryptophanate, 15 mg light yellow solid; $[\alpha]^{25}_{D}$ -6.13 (C 0.10, CH_3OH); UV(MeOH) λmax (log ε): 225, 290 nm; IR(KBr)νmax 3380, 3262, 1745, 1731, 1645, 1535 cm^{-1}; HRMS (ESI-TOF) (m/z): calcd for $C_{26}H_{30}N_2O_4$ ($[M + H]^+$), 435.2278, found, 435.2244; ^{1}H and ^{13}C-NMR data, see Table 1.

Total synthesis of Misszrtine A (1):

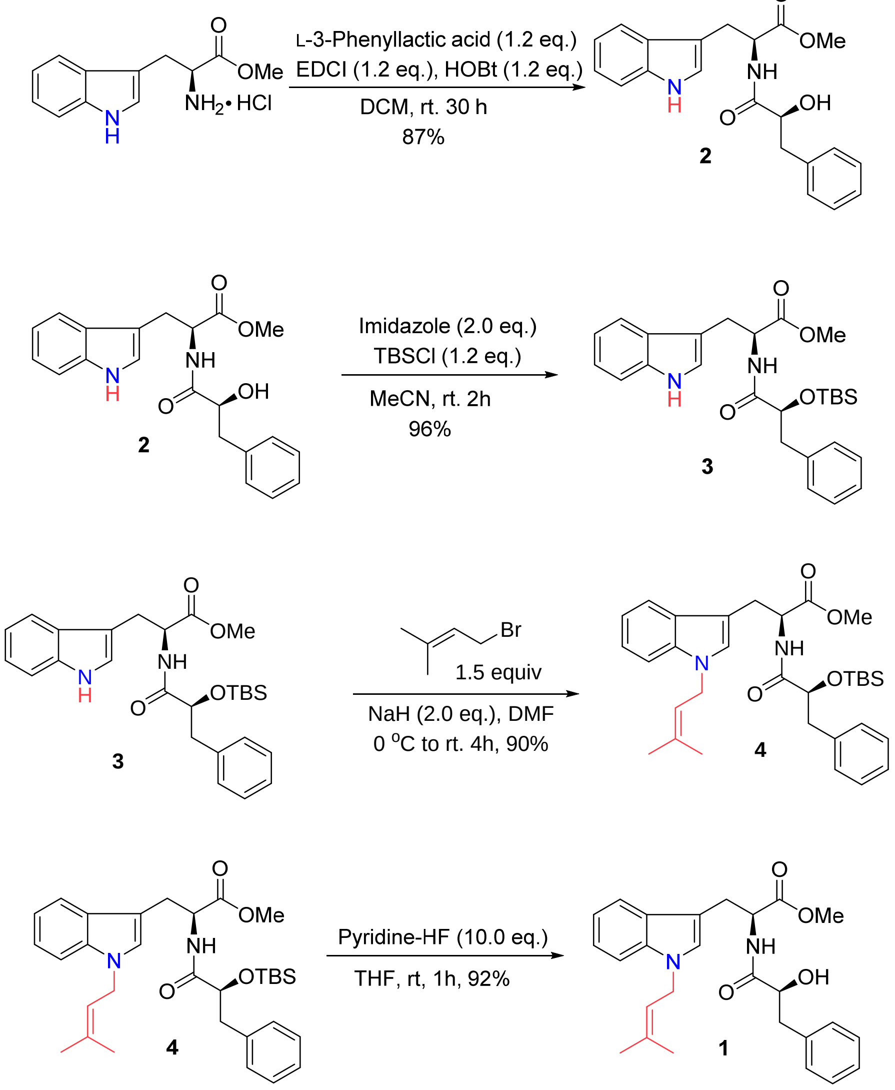

Figure S1. UV spectrum of Misszrtine A (1) in MeOH.

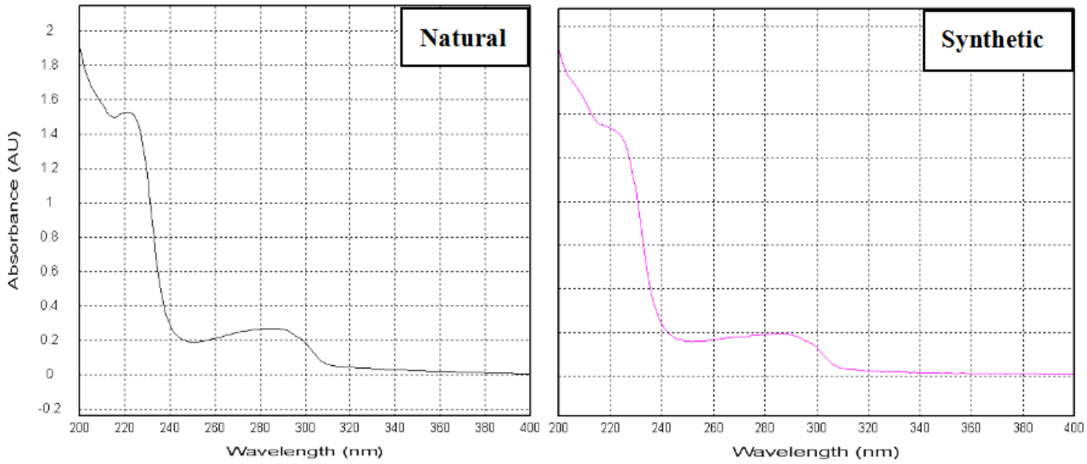

Figure S2. CD spectrum of MisszrtlideA (1) in MeOH.

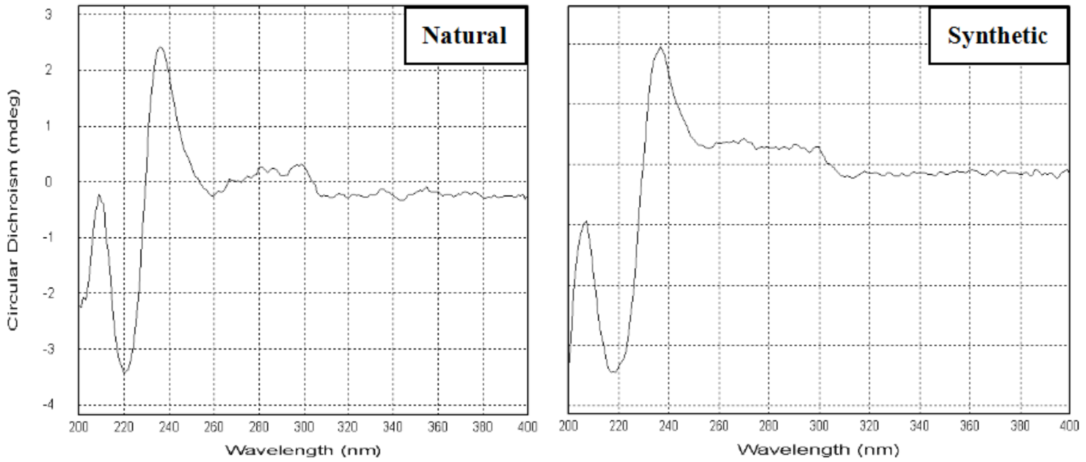

NMR spectra:

Figure S3. ^{1}H NMR spectrum of natural Misszrtine A (1) in $CDCl_3$.

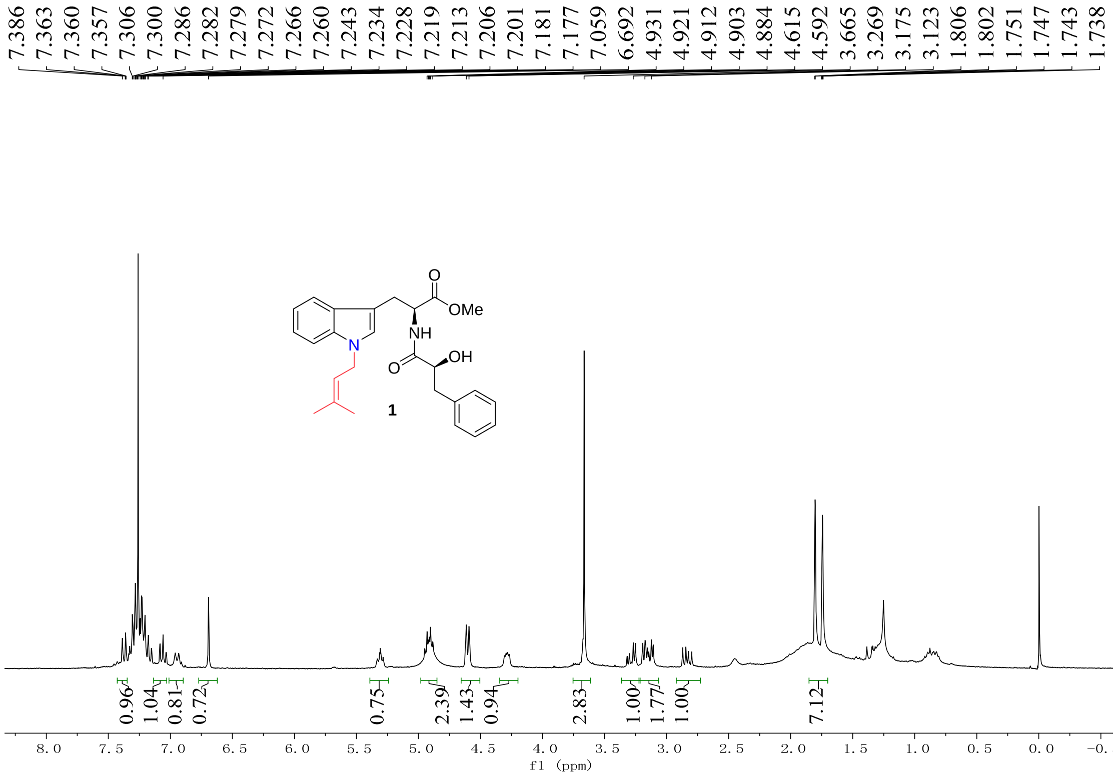

Figure S4. ^{13}C NMR spectrum of natural Misszrtine A (**1**) in $CDCl_3$.

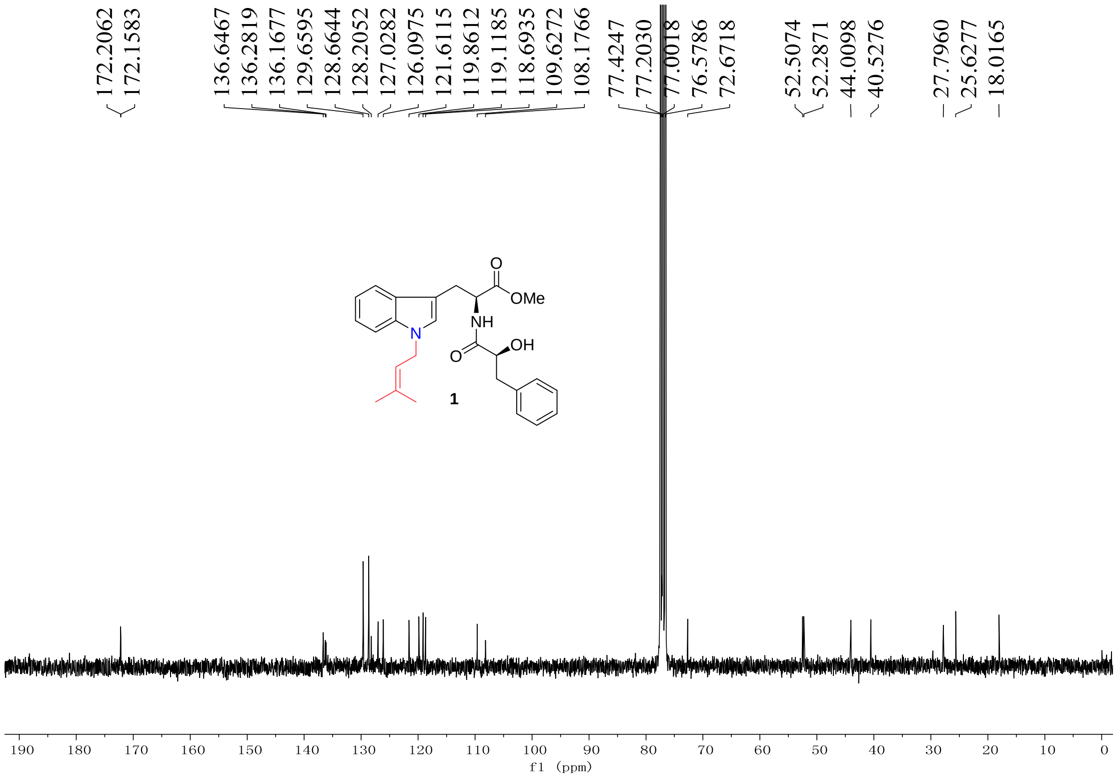

Figure S5. DEPT135 spectrum of natural Misszrtine A (1) in $CDCl_3$.

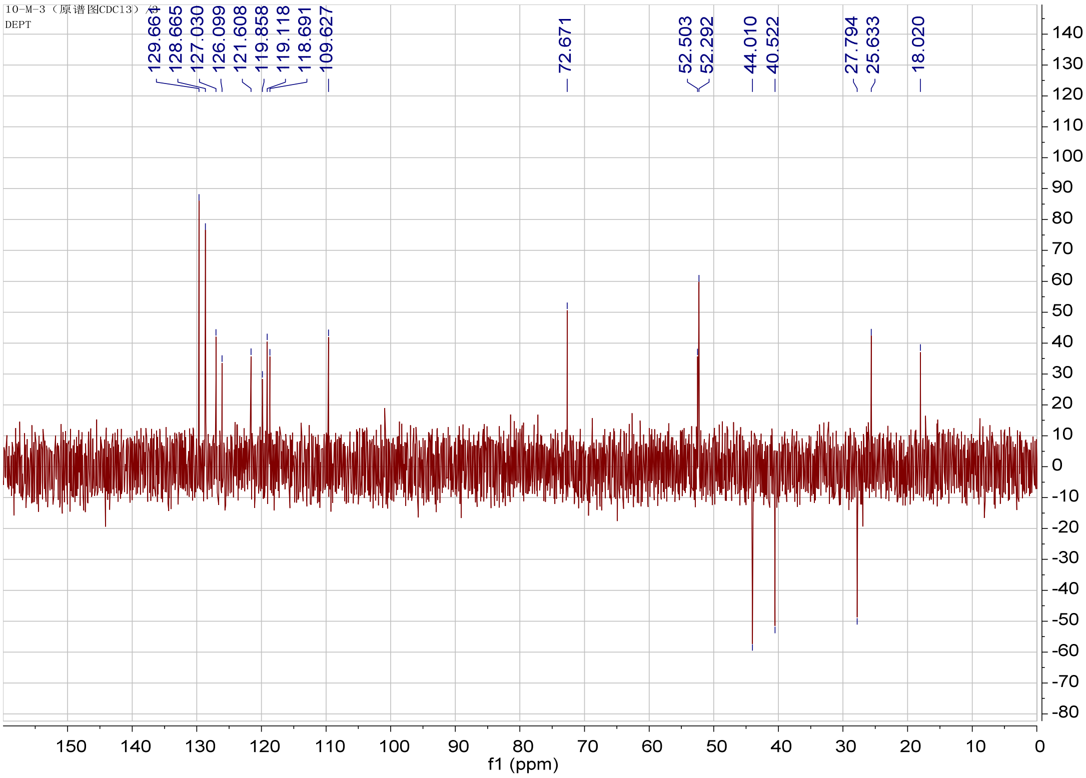

Figure S6. COSY spectrum of natural Misszrtine A (1) in $CDCl_3$.

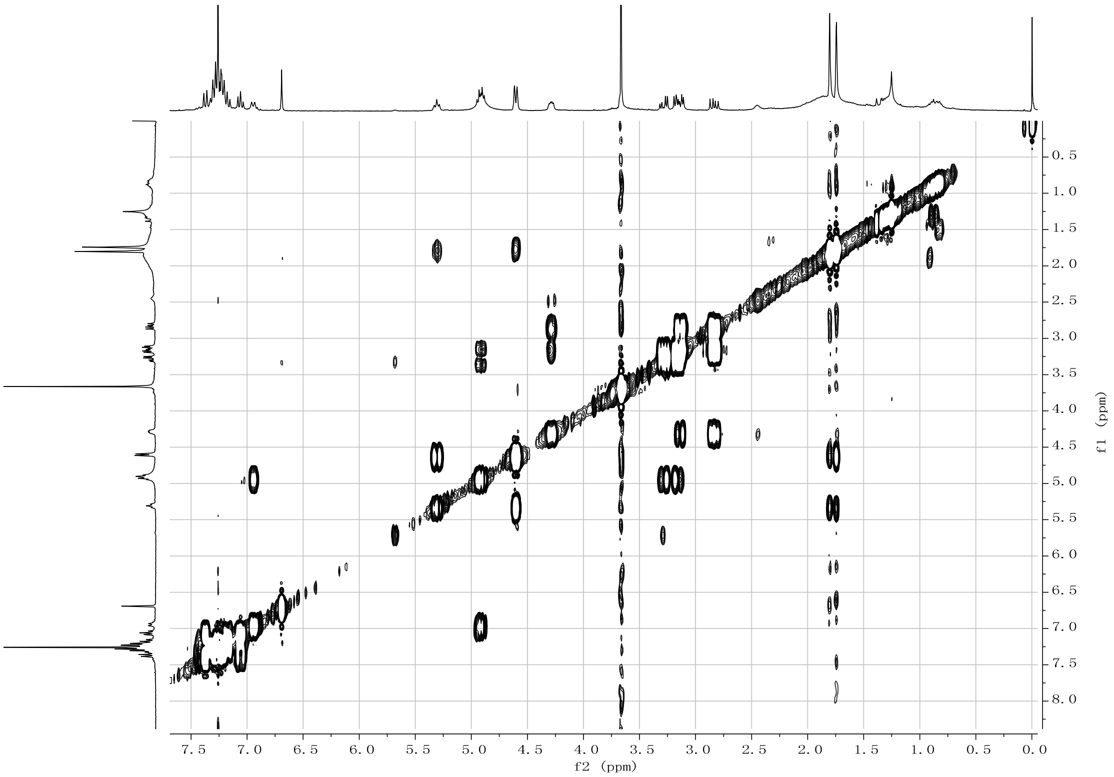

Figure S7. HSQC spectrum of natural Misszrtine A (1) in $CDCl_3$.

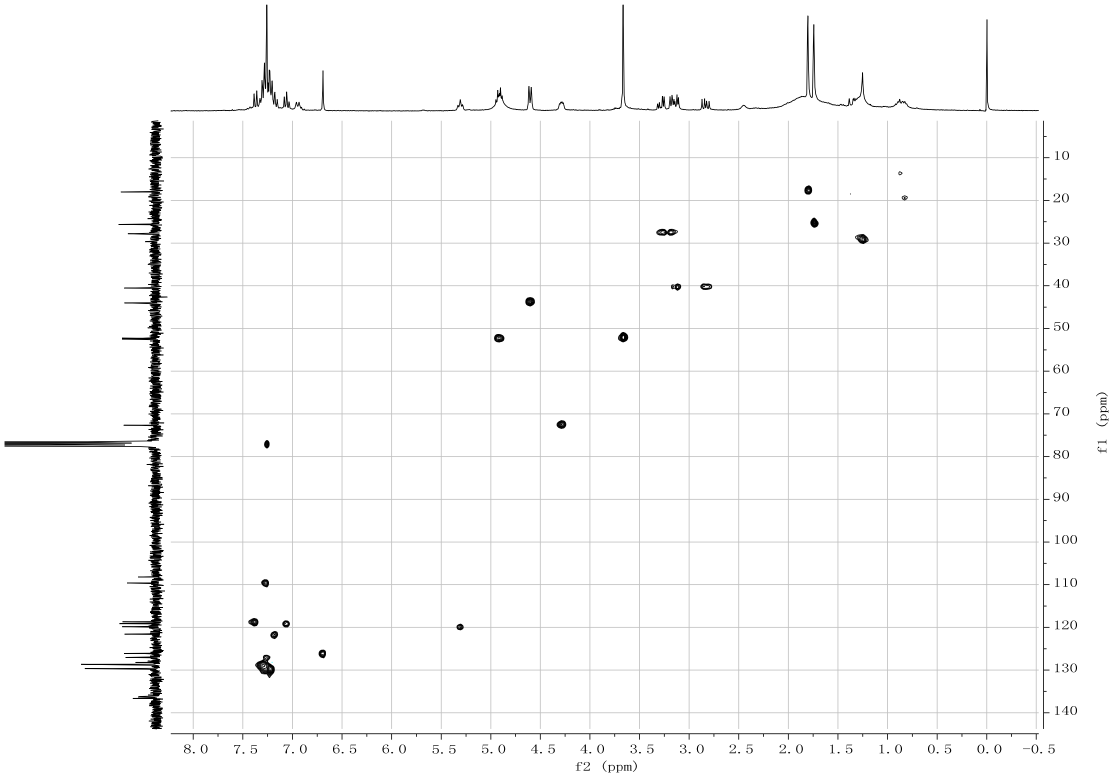

Figure S8. HMBC spectrum of natural Misszrtine A (1) in $CDCl_3$.

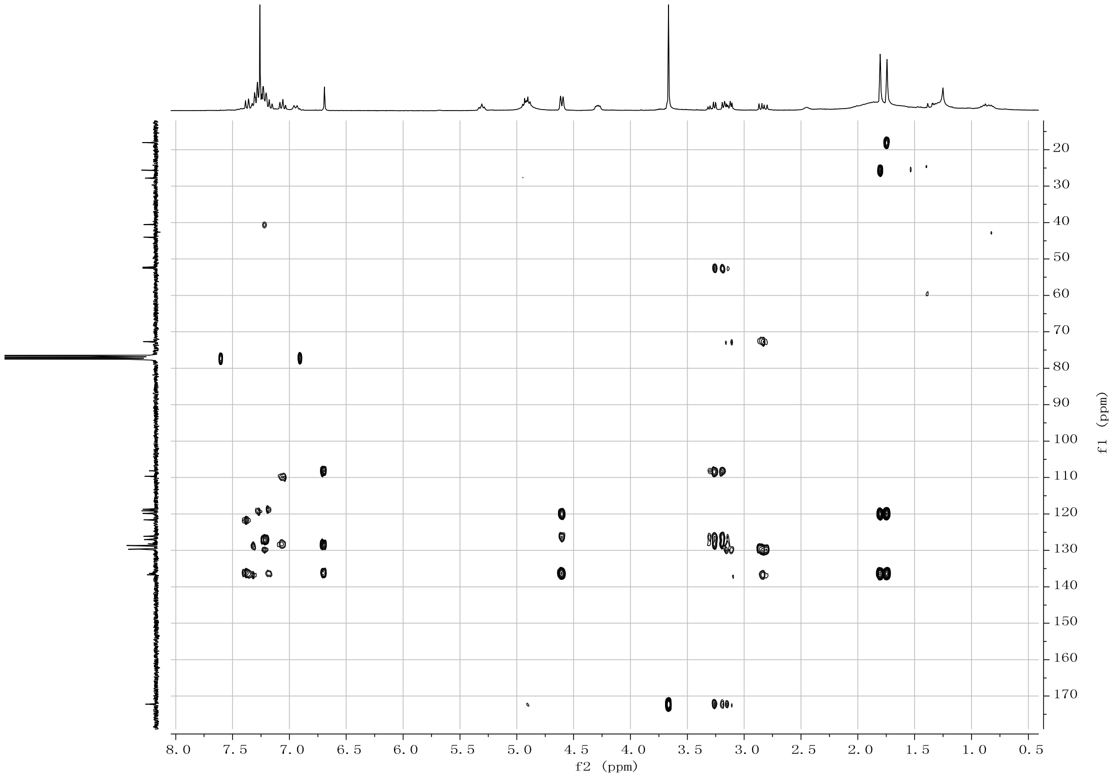

Figure S9. NOESY spectrum of natural Misszrtine A (1) in $CDCl_3$.

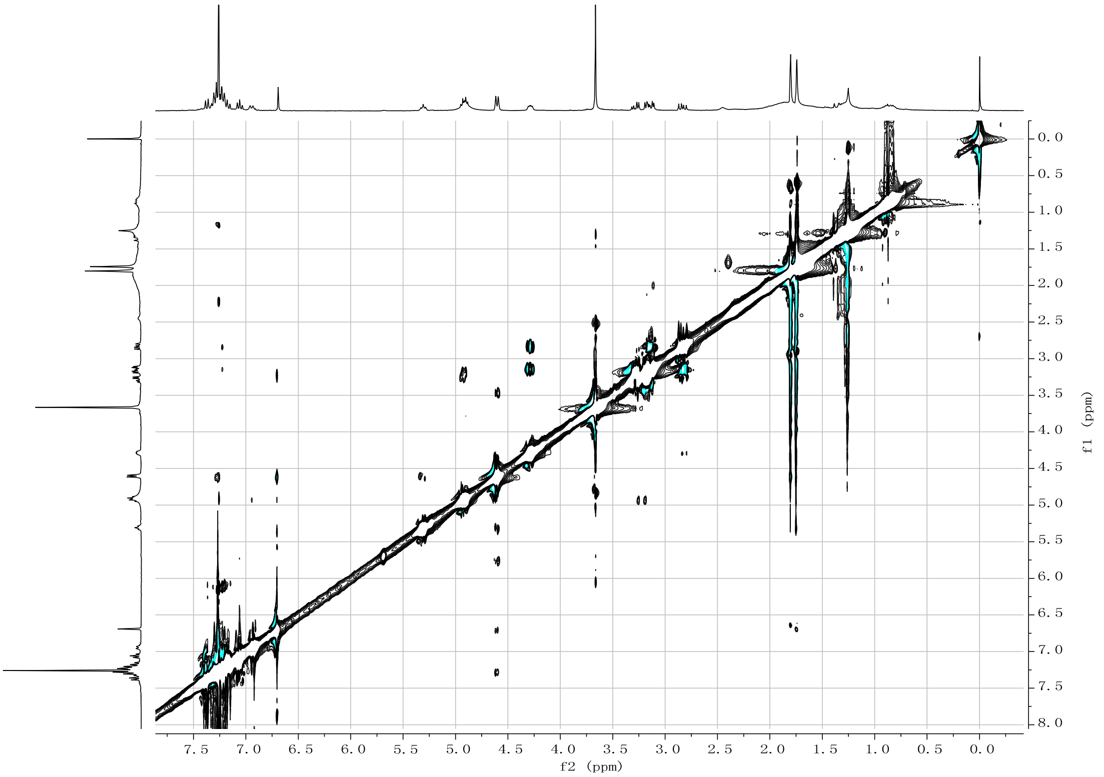

Figure S10. ^{1}H NMR spectrum of 2 in $CDCl_3$

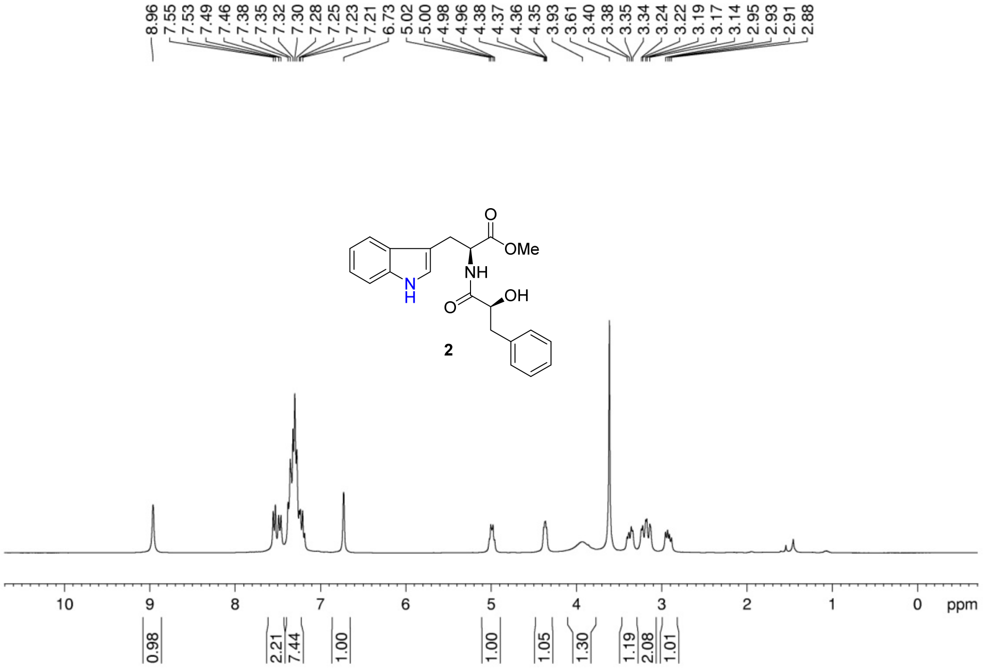

Figure S11. ^{13}C NMR spectrum of 2 in $CDCl_3$.

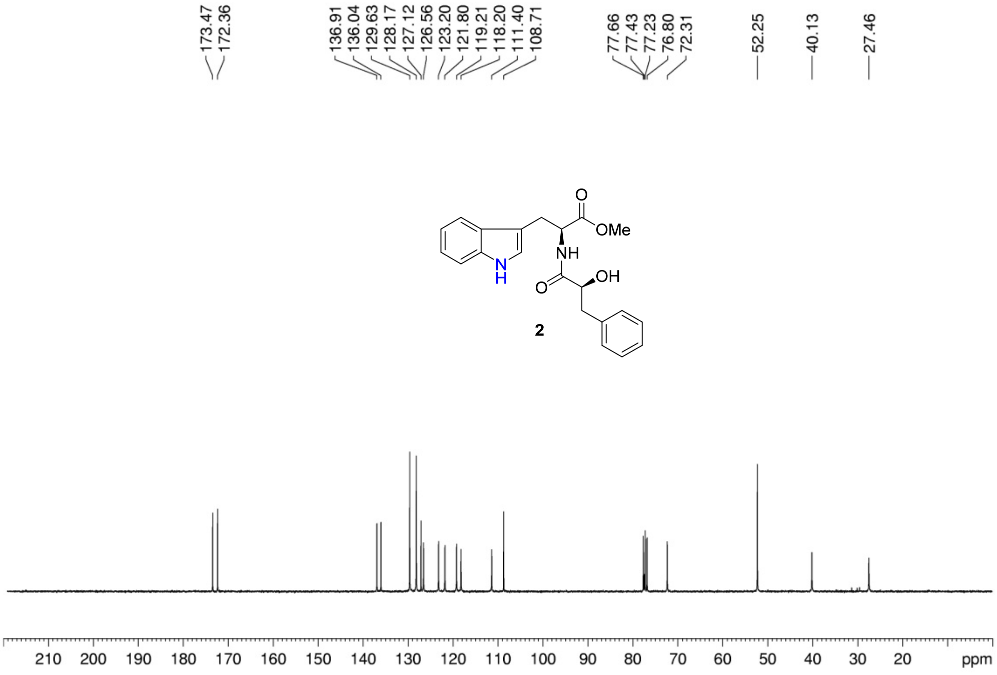

Figure S12 ^{1}H NMR spectrum of synthesized Misszrtine A (1) in $CDCl_3$.

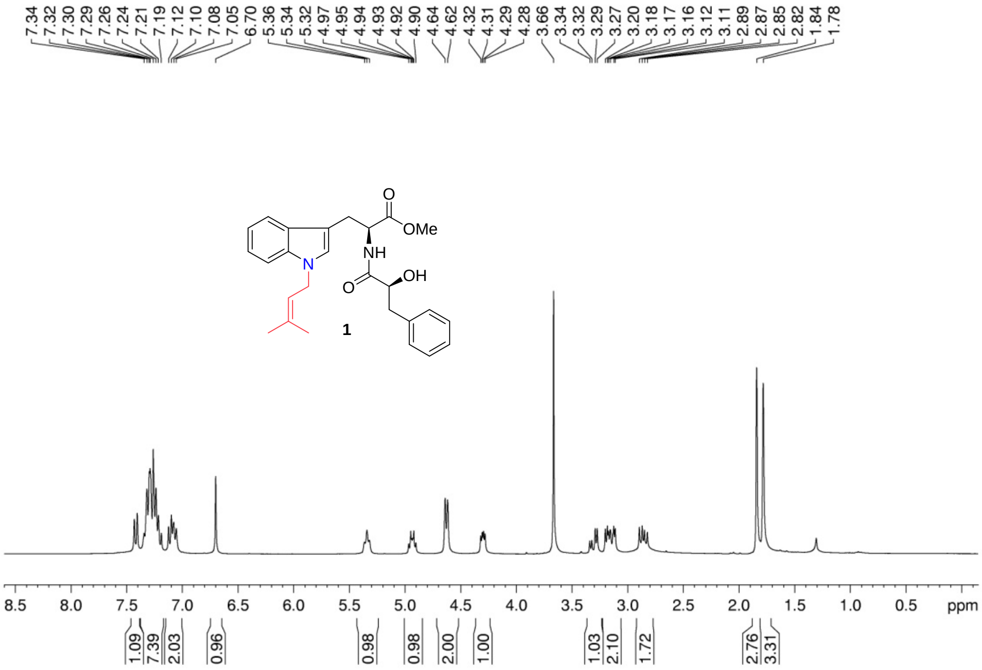

Figure S13 ^{13}C NMR spectrum of synthesized Misszrtine A (1) in $CDCl_3$.

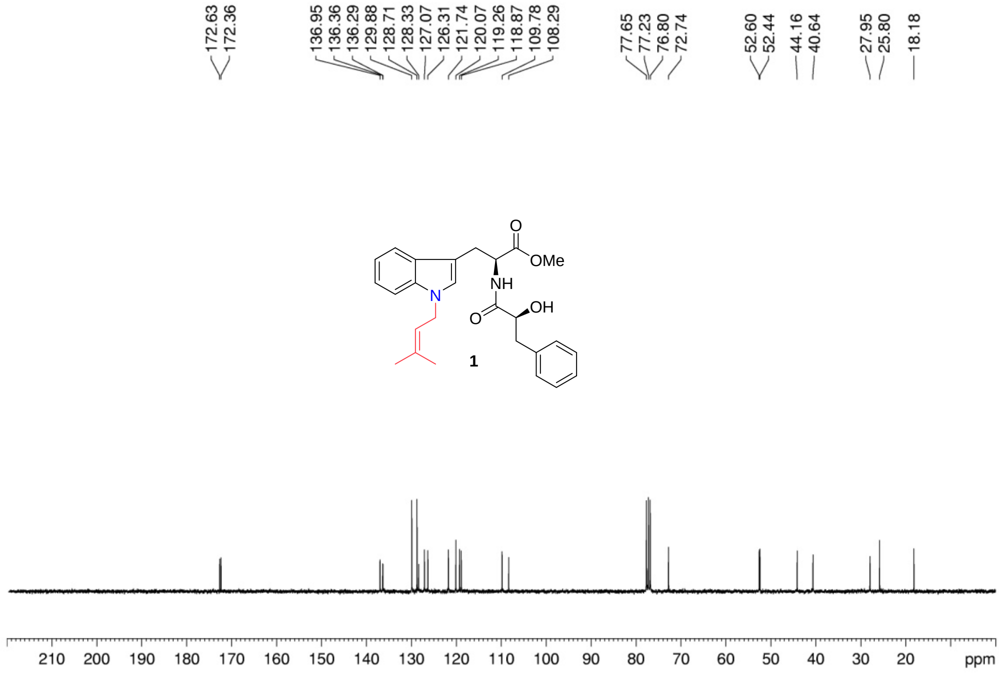

Supplement: Supplementary file 1 [file Presentation_1.pdf]
